# Asiatic Acid Prevents Cognitive Deficits by Inhibiting Calpain Activation and Preserving Synaptic and Mitochondrial Function in Rats with Kainic Acid-Induced Seizure

**DOI:** 10.3390/biomedicines9030284

**Published:** 2021-03-10

**Authors:** Cheng-Wei Lu, Tzu-Yu Lin, Tai-Long Pan, Pei-Wen Wang, Kuan-Ming Chiu, Ming-Yi Lee, Su-Jane Wang

**Affiliations:** 1 Department of Anesthesiology, Far-Eastern Memorial Hospital, New Taipei 22060, Taiwan; drluchengwei@gmail.com (C.-W.L.); drlin1971@gmail.com (T.-Y.L.); 2 Department of Mechanical Engineering, Yuan Ze University, Taoyuan 32003, Taiwan; 3 School of Traditional Chinese Medicine, Chang Gung University, Taoyuan 33302, Taiwan; pan@mail.cgu.edu.tw; 4 Liver Research Center, Chang Gung Memorial Hospital, Taoyuan 33375, Taiwan; 5 Department of Medical Research, China Medical University Hospital, China Medical University, Taichung 40447, Taiwan; pwwang5105@gmail.com; 6 Division of Cardiovascular Surgery, Cardiovascular Center, Far-Eastern Memorial Hospital, New Taipei 22060, Taiwan; chiu9101018@gmail.com (K.-M.C.); mingyi.lee@gmail.com (M.-Y.L.); 7 Department of Nursing, Oriental Institute of Technology, New Taipei 22060, Taiwan; 8 Department of Photonics Engineering, Yuan Ze University, Taoyuan 32003, Taiwan; 9 School of Medicine, Fu Jen Catholic University, No.510, Zhongzheng Rd., Xinzhuang Dist., New Taipei City 24205, Taiwan; 10 Research Center for Chinese Herbal Medicine, College of Human Ecology, Chang Gung University of Science and Technology, Taoyuan 33303, Taiwan

**Keywords:** asiatic acid, epilepsy, cognitive dysfunction, kainic acid, synaptic proteins, mitochondrion proteins

## Abstract

Cognitive impairment is not only associated with seizures but also reported as an adverse effect of antiepileptic drugs. Thus, new molecules that can ameliorate seizures and maintain satisfactory cognitive function should be developed. The antiepileptic potential of asiatic acid, a triterpene derived from the medicinal herb *Centella asiatica*, has already been demonstrated; however, its role in epilepsy-related cognitive deficits is yet to be determined. In this study, we evaluated the effects of asiatic acid on cognitive deficits in rats with kainic acid (KA)-induced seizure and explored the potential mechanisms underlying these effects. Our results revealed that asiatic acid administrated intraperitoneally 30 min prior to KA (15 mg/kg) injection ameliorated seizures and significantly improved KA-induced memory deficits, as demonstrated by the results of the Morris water maze test. In addition, asiatic acid ameliorated neuronal damage, inhibited calpain activation, and increased protein kinase B (AKT) activation in the hippocampus of KA-treated rats. Asiatic acid also increased the levels of synaptic proteins and the number of synaptic vesicles as well as attenuated mitochondrial morphology damage in the hippocampus of KA-treated rats. Furthermore, proteomic and Western blot analyses of hippocampal synaptosomes revealed that asiatic acid reversed KA-induced changes in mitochondria function-associated proteins, including lipoamide dehydrogenase, glutamate dehydrogenase 1 (GLUD1), ATP synthase (ATP5A), and mitochondrial deacetylase sirtuin-3 (SIRT3). Our data suggest that asiatic acid can prevent seizures and improve cognitive impairment in KA-treated rats by reducing hippocampal neuronal damage through the inhibition of calpain activation and the elevation of activated AKT, coupled with an increase in synaptic and mitochondrial function.

## 1. Introduction

Epilepsy is a chronic neurological disorder affecting approximately 70 million people worldwide [1]. This disease is often accompanied by numerous comorbidities, particularly learning and memory deficits [2,3,4]. In addition, the long-term use of anti-epileptic drugs (AEDs) can cause memory impairment in patients with epilepsy [5,6]. However, current antiepileptic medications only help with seizures and do not improve cognitive dysfunction. Therefore, novel therapeutic drugs that can treat epilepsy and maintain favorable cognitive function must be developed.

**Figure 1:**
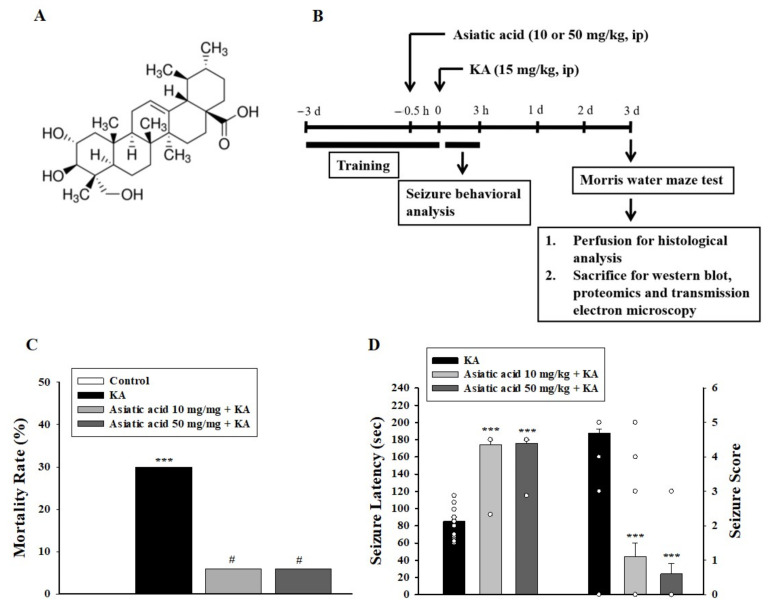
Asiatic acid pretreatment attenuated seizures in rats subjected to KA (kainic acid). (**A**) Chemical structure of asiatic acid. (**B**) Schematic timeline of the experimental protocol. (**C**) Mortality rate and (**D**) seizure latency and seizure score in the presence of asiatic acid versus the animals that were injected only with KA. *** *p* < 0.001 (vs. control); # *p* < 0.001 (asiatic acid + KA vs. KA); *n* = 14–20 rats per group.

Recently, medicinal plant-derived substances have been highlighted because of their abilities to penetrate the blood–brain barrier, modulate neuronal activities, and prevent neuronal damage [7,8]. Asiatic acid (Figure 1A) is a triterpene derived from the medicinal herb *Centella asiatica* (L.) Urban (*Apiaceae*) [9]. Asiatic acid has been extensively studied using various in vitro and in vivo animal models, and it exhibits strong neuroprotective, anticonvulsive, and cognition-enhancing properties, as well as improves conditions such as cerebral ischemia, Alzheimer’s disease, Parkinson’s disease, and spinal cord injury [10,11,12,13,14,15,16,17,18]. Furthermore, studies have reported the low toxicity of asiatic acid and its ability to penetrate through the blood–brain barrier (BBB) [19,20]. Therefore, asiatic acid can have therapeutic potential for brain disorders, including epilepsy.

Epilepsy can be reproduced using the appropriate animal models. For example, the systemic administration of kainic acid (KA), a glutamate analog, can induce recurrent behavioral seizures, deficient cognitive functions, and disruptive morphological changes in different areas of the brain. In particular, the hippocampus has been established as a site of damage following KA administration; this damage causes cognitive dysfunction [21,22,23]. These pathological changes induced by KA share similarities with those found in patients with epilepsy [24,25]. Thus, the KA-induced seizure animal model is widely used for studying epilepsy [26]. Asiatic acid can protect against KA-induced seizures [15]; however, its preventive effects on KA-induced cognitive impairment have not been determined. In this study, we investigated the effects of asiatic acid on KA-induced cognitive deficits in rats and explored its potential underlying mechanism.

## 2. Materials and Methods

### 2.1. Chemicals

Asiatic acid (purity > 99%, Aktin, A98688, Chengdu, China), KA (Sigma-Aldrich, K0250, St. Louis, MO, USA), dimethylsulfoxide (DMSO, Sigma-Aldrich, D8418, St. Louis, MO, USA), chloral hydrate, neutral red (Sigma-Aldrich, N2880, St. Louis, MO, USA), and general reagents were purchased from Sigma-Aldrich (St. Louis, MO, USA); Fluoro-Jade B (FJB, Histo-Chem Inc., 1FJB, Jefferson, AR, USA) was obtained from Millipore (Temecula, CA, USA).

### 2.2. Animals

A total of 68 adult male Sprague-Dawley rats (*n* = 64) weighing between 150 and 200 g were purchased from BioLASCO (Taipei, Taiwan). Animals were provided food and water ad libitum and housed under a 12-h light/12-h dark cycle. Experiments were performed in accordance with approved animal protocols and guidelines established by the Animal Care Committee of Fu Jen Catholic University (Institutional Animal Care and Use Committee (IACUC) at the Fu Jen Catholic University, No. AE10561, 03 May 2017).

### 2.3. Experimental Design

Figure 1B shows the experimental design. Rats underwent the Morris water maze task for 3 days. Subsequently, they were randomly divided into four groups: DMSO-treated group (control), KA only-treated group, 10 mg/kg asiatic acid + KA group, and 50 mg/kg asiatic acid + KA group. KA (15 mg/kg) dissolved in 0.9% NaCl (pH 7.0) was intraperitoneally (i.p.) injected to induce seizures. Asiatic acid dissolved in 1% DMSO was administered (i.p.) 30 min prior to KA injection. After KA injection, seizure activity was rated during a 3-h period according to the Racine scale [27]. The time of seizure initiation and seizure scores were analyzed. After the Morris water maze test, rats were deeply anesthetized and sacrificed on the third day. To examine the immunohistochemistry of the hippocampus, rats were subjected to cardiac perfusion with normal saline, followed by 4% paraformaldehyde (PFA, Sigma-Aldrich, P6148, St. Louis, MO, USA). The brains were post-fixed with 4% PFA for 3 h at room temperature and then cryoprotected in 10% sucrose solution for up to 5 days at 4 °C. Subsequently, 30-μm coronal sections of the fixed brain were cut using a standard microtome, and 5-in-1 series of brain sections throughout the hippocampus were collected in wells containing antifreeze (20% glycerol and 30% ethylene glycol) solution and stored at −20 °C. In addition, another set of animals (*n* = 20) were deeply anesthetized and rapidly decapitated on the third day after administering KA. Fresh brain tissues from both the hippocampi were collected for synaptosomal preparation and Western blot analysis. The KA dose and administration schedule were chosen based on previous experiments [28,29,30].

### 2.4. Morris Water Maze Test

The Morris water maze test was performed to evaluate the spatial learning and memory of rats, as described previously [31]. During the first 3 days, rats were trained to find a platform (10 cm in diameter) hidden 2 cm below the water surface level in a circular pool (200 cm in diameter and 50 cm in depth) at a fixed location. The apparatus was filled with water (22 °C ± 1 °C) to a depth of approximately 35 cm. The pool was virtually divided into four equal-size quadrants. On each day of training, a rat was placed in a different quadrant facing the pool wall and was trained to find the escape platform within 120 s. If the platform was not found within 120 s, the rat was guided to the platform by the experimenter. Each rat was allowed to remain on the platform for 30 s during each trial. Each rat performed four training trials daily for 3 consecutive days, with an interval of approximately 60 s between trials. The latency (time taken to find the escape platform) and the swimming distances were recorded using a video tracking system (Version 1.17, SINGA Technology Corporation, Taipei, Taiwan).

### 2.5. Neutral Red and FJB Staining

Neutral red staining was performed to examine the general histology. The brain sections were mounted on gelatinized slides and stained with 1% neutral red. Each stained section was observed using a light microscope to assess the degree of neuronal loss within the hippocampus. FJB staining of the degenerating neurons was performed on another set of brain sections, as described previously [31]. Briefly, the sections were mounted on gelatinized slides and immersed in 100% ethanol (3 min), 70% ethanol (2 min), and distilled water (2 min). Subsequently, the sections were incubated in 0.06% potassium permanganate (J.T. Baker Inc., 3227-01, Phillipsburg, NJ, USA) for 15 min, washed twice in distilled water, and immersed in FJB solution (0.001% FJB/0.1% acetic acid) for 30 min in darkness. The potassium permanganate pretreatment not only confers considerable resistance to fading, but also minimizes background staining. After washing three times in distilled water, the slides were air dried in the dark for 20 min, dehydrated in xylene (9490-03), and coverslipped using dibutyl phthalate in xylene medium. Images were acquired using an upright fluorescence microscope (Zeiss Axioskop 40, Göttingen, Lower Saxony, Germany). Leica 4X or 10X objective lenses with a numerical aperture (NA) of 0.1 or 0.25 were used in this study (Wetzlar, Germany). To compare neuronal death among the experimental groups, the numbers of surviving neurons and Fluoro-Jade B-positive cells was counted in a 255 × 255 μm^2^ area of the hippocampal Cornu Ammonis 1 (CA1) and CA3 in 6–8 randomly chosen sections from each animal and averaged for each animal using Image J by an examiner blind to the experimental conditions. Results are expressed as the mean ± standard error of mean (SEM) of the labeled cells per 0.1 mm^2^.

### 2.6. Synaptosomal Preparation and Transmission Electron Microscopy

Synaptosomes were prepared from male Sprague-Dawley rats as described previously [30,32]. Briefly, rats (*n* = 3/group) were sacrificed through rapid decapitation, and the hippocampus was removed and homogenized in 0.32 M sucrose. After centrifugation (5000 rpm for 10 min), the supernatant was placed into Percoll (Sigma-Aldrich, P1644, St. Louis, MO, USA) discontinuous gradients (3%, 10% and 23%) and centrifuged at 16,500 rpm for 7 min. The synaptosomal fraction between 10% and 23% Percoll bands was collected and centrifuged for 10 min at 15,000 rpm. Synaptosomes were fixed in 4% PFA and 2.5% glutaraldehyde for 1 day. The fixed hippocampal synaptosomes were washed in PBS, postfixed in 1% osmium tetraoxide for 2 h, dehydrated, and embedded in epoxy resin. Subsequently, 70-nm-thick sections were cut using an ultramicrotome (EM UC7, Leica Microsystems, Wetzlar, Germany). The ultrastructure of the hippocampal synaptosome was observed under a transmission electron microscope (JEM-1400, JEOL, Tokyo, Japan).

### 2.7. Proteomics

The two-dimensional electrophoresis (2-DE) procedure is described in a previous study [33]. Briefly, protein extracts (180 μg) were applied to ImmobilineDrystrip (pH 4–7, 18-cm IPG strip, GE Healthcare, Chicago, IL, USA) and separated on the IPGphor III System to evaluate the first dimension. The 2-DE procedure was performed using 10% acrylamide gels (Bio-Rad, Hercules, CA, USA), and the protein images were visualized through silver staining. All gels were scanned and quantified using Prodigy SameSpots software (Nonlinear Dynamics, Newcastle, UK). Each spot intensity volume (%) was determined through the background subtraction technique and the total spot volume normalization method for comparison among groups. More than 2.0-fold alterations at 95% confidence intervals (*p* < 0.05) were considered statistically significant. All experiments were repeated three times to confirm reproducibility.

Spots of interest were excised, and in-gel digested with trypsin as described previously [34]. After digestion, tryptic peptides were acidified with 0.5% trifluoroacetic acid and loaded onto an MTP AnchorChip™ 600/384 TF (Bruker-Daltonik, Bremen, Germany). Mass spectrometry analysis was performed using an Ultraflex™ MALDI-TOF mass spectrometer (Bruker-Daltonik, Bremen, Germany). Monoisotopic peptide masses were assigned and used for database searches in the MASCOT search engine (Matrix Science, London, UK). Search parameters were set as follows: a maximum allowed peptide mass error of 50 ppm and the consideration of one incomplete cleavage per peptide.

### 2.8. Biological Network Analysis Using MetaCore™

MetaCore™ software (version 5.2 build 17389, GeneGo, St. Joseph, MI, USA) was utilized to elucidate the ontological classes and relevant pathways represented by genes identified in 2-DE and peptide mass fingerprinting. We used two algorithms for the network analysis: (i) the analysis network algorithm, to deduce the scoring processes regulated by the differentially expressed proteins; and (ii) the shortest path algorithm, to build a network consisting of the smallest possible number of direct interactions between the differentially expressed proteins. The statistical relevance of the ontological matches was calculated as the *p* value [35].

### 2.9. Western Blotting

The sample preparation procedure and conditions for Western blot analysis are described previously [36]. In brief, the hippocampi or hippocampal synaptosomes were homogenized, and equal amounts of protein were subjected to sodium dodecylsulfate polyacrylamide gel electrophoresis and blotted onto nitrocellulose membranes. Subsequently, membranes were first incubated for 1 h in 5% non-fat milk and then overnight at 4 °C with primary antibodies. The antibodies used were anti-calpain 1 (1:1000, Abcam, ab28258, Cambridge, UK), anti-calpain 2 (1:800, Millipore, MAB3083, Temecula, CA, USA), anti-calpastatin (1:300, Proteintech, 12250-1-AP, Rosemont, IL, USA), anti-protein kinase B (AKT, 1:10,000, Cell Signaling, #9272, Beverly, MA, USA), anti-pAKT (1:10000, Cell Signaling, #9271, Beverly, MA, USA), anti-synaptophysin (1:100,000, Cell Signaling, #36406, Beverly, MA, USA), anti-synaptotagmin (1:1000, Abcam, ab13259, Cambridge, UK), anti-synaptobrevin (1:800, Abcam, ab18013, Cambridge, UK), anti-synapsin I (1:100,000, Cell Signaling, #5297, Beverly, MA, USA), anti-synaptosomal-associated protein 25 kDa (SNAP 25, 1:6000, Abcam, ab41455, Cambridge, UK), anti-postsynaptic density protein 95 (PSD 95, 1:600, Abcam, ab2723, Cambridge, UK), anti-lipoamide dehydrogenase (1:20,000, Abcam, ab133551, Cambridge, UK), anti-glutamate dehydrogenase 1 (GLUD1, 1:1000, Invitrogen, #PA5-28301, Waltham, MA, USA), anti-mitochondrial membrane ATP synthase (ATP5A, 1:3000, Abcam, ab14748, Cambridge, UK), anti-mitochondrial deacetylase sirtuin-3 (SIRT3, 1:800, Abcam, ab270523, Cambridge, UK), and anti-β-actin (1:1000, Cell Signaling, #3700, Beverly, MA, USA), which served as a control of the protein load. The membranes were washed three times (Tris-buffered saline, TBS, 10 min each), incubated with horseradish peroxidase-conjugated secondary antibodies (1:2000, Gentex, GTX213110-01, GTX213111-01, Zeeland, MI, USA) for 1 h, and washed with TBS three times (10 min each), followed by detection using the enhanced chemiluminescence system (Amersham Biosciences Corp., Amersham, Buckinghamshire, UK) and quantification using Image J analysis software (Synoptics, Cambridge, UK).

### 2.10. Statistical Analyses

The results are expressed as the mean ± SEM and were analyzed using one-way analysis of variance (ANOVA), followed by Tukey’s post-hoc test to determine the difference. Differences were considered statistically significant at *p* < 0.05.

## 3. Results

### 3.1. Asiatic Acid Reduced the Mortality Rate and Seizures in Rats Receiving KA

KA (15 mg/kg, i.p.) induced seizures in 91% of injected rats, with a mortality rate of 30%. Asiatic acid (10 and 50 mg/kg) administered (i.p.) 30 min prior to KA administration significantly reduced the mortality rate to 6% (Figure 1C). In addition, we observed that asiatic acid pretreatment delayed seizure initiation (*F* (2,42) = 103.6, *p* < 0.001; Figure 1D) and reduced the severity of seizures compared with only KA treatment (*F* (2,51) = 42.3, *p* < 0.001; Figure 1D).

### 3.2. Asiatic Acid Improved Learning and Memory Deficits in Rats with KA-Induced Seizures

**Figure 2:**
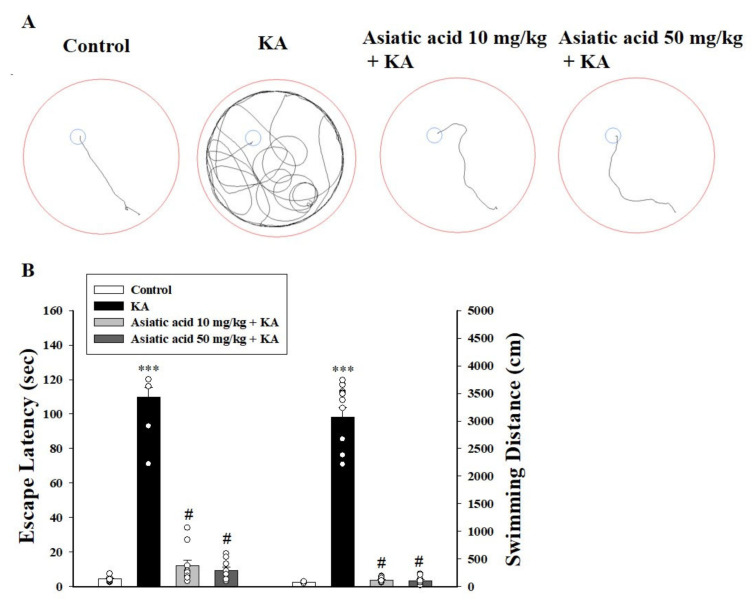
Effect of asiatic acid pretreatment on spatial learning and memory in rats with KA (kainic acid)-induced seizures. (**A**) Swimming tracks of rats in the Morris water maze test. (**B**) Mean escape latency to reach the platform and total distance traveled to reach the platform. *** *p* < 0.001 (vs. control); # *p* < 0.001 (asiatic acid + KA vs. KA); *n* = 8–13 rats per group.

To investigate the effect of asiatic acid on learning and memory, rats were subjected to the Morris water maze test (Figure 2A). The time taken to locate the escape platform and the total distance traveled are shown in Figure 2B. We observed that the KA-treated rats required a longer time to reach the platform than did the control rats (*p* < 0.001). Pretreatment of the KA-treated rats with asiatic acid significantly shortened the escape time compared with rats with KA treatment alone (*F* (3,45) = 224.9, *p* < 0.001). The KA group showed an increase in the total distance traveled to reach the platform compared with the control group (*p* < 0.001). Pretreatment with asiatic acid significantly reduced the total distance traveled compared with KA treatment alone (*F* (3,37) = 202.9, *p* < 0.001; Figure 2B). No difference in the total distance traveled was noted between the groups treated with asiatic acid and the control group (*p* = 0.99).

### 3.3. Asiatic Acid Attenuated Neuronal Damage in the Hippocampus of Rats with KA-Induced Seizures

**Figure 3:**
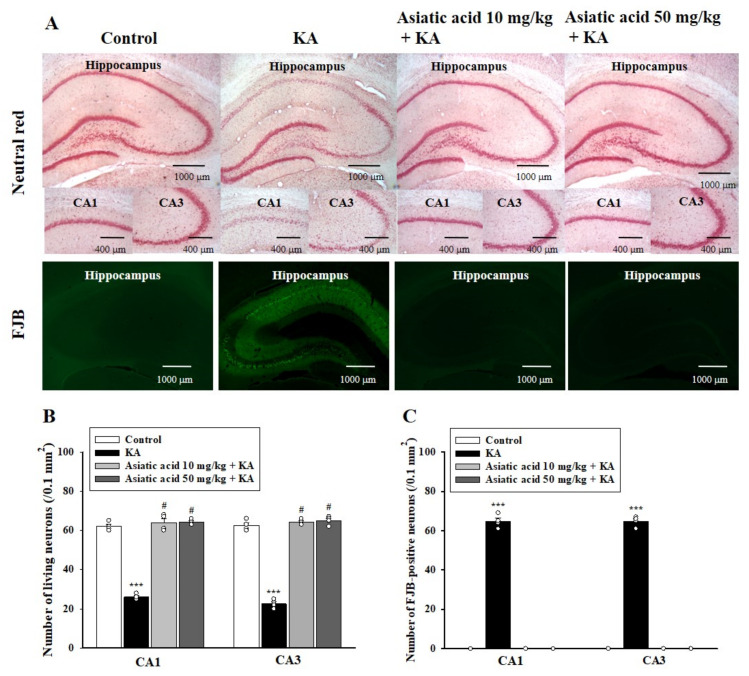
Effects of asiatic acid pretreatment on neuronal damage in the hippocampus of rats with KA-induced seizures. (**A**) Representative images showing neutral red and FJB (Fluoro-Jade B) staining in the hippocampus. The number of surviving neurons (**B**) and FJB-positive neurons (**C**) in the hippocampal CA1 and CA3 regions was counted. *** *p* < 0.001 (vs. control); # *p* < 0.001 (asiatic acid + KA vs. KA); *n* = 4 rats per group. Scale bar: 400–1000 μm.

Hippocampal neuronal damage induced by KA can lead to cognitive impairment [37]. Therefore, we evaluated the effect of asiatic acid on neuronal damage after rats underwent the Morris water maze test. As shown in Figure 3A, cell viability in the hippocampus was examined through neutral red staining. Cell viability in the CA1 and CA3 areas of the hippocampus 3 days after KA treatment was significantly lower than that in the control group. Cell survival in the CA1 and CA3 areas of the hippocampus was significantly higher in the asiatic acid pretreatment group than in the KA-only treated group (CA1, *F* (3,12) = 219.5, *p* < 0.001; CA3, *F* (3,12) = 376.9, *p* < 0.001; Figure 3A,B). The protective effect of asiatic acid was also observed through FJB staining, which shows cells undergoing neurodegeneration. As shown in Figure 3A, no cells were stained with FJB in the control rats. By contrast, abundant FJB-positive cells were observed in the CA1 and CA3 regions of the KA-treated rats. In rats pretreated with asiatic acid, no FJB-positive cells were found in the CA1 and CA3 regions. These findings were corroborated by one-way ANOVA results that revealed that KA administration significantly increased the number of FJB-positive cells and that asiatic acid significantly suppressed this effect (CA1, *F* (3,12) = 1536.2, *p* < 0.001; CA3, *F* (3,12) = 2424.6, *p* < 0.001; Figure 3C). However, no such differences were noted between groups treated with asiatic acid and the control group (*p* = 1).

### 3.4. Asiatic Acid Suppressed Calpain Activation and AKT Inactivation in the Hippocampus of Rats with KA-Induced Seizures

**Figure 4:**
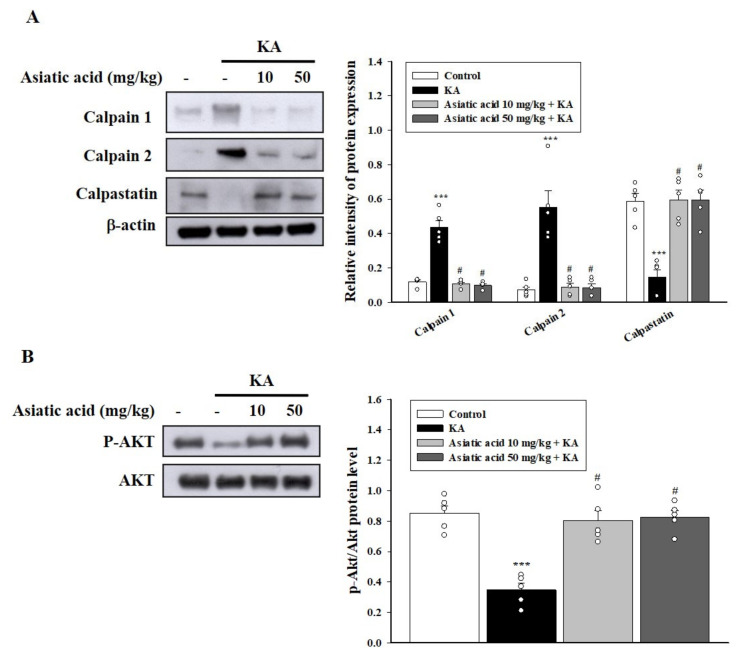
Effects of asiatic acid pretreatment on the levels of calpain, calpastatin, and p-AKT (protein kinase B) in the hippocampus of rats with KA-induced seizures. (**A**,**B**) Western blot showing the expression levels of calpain, calpastatin, and p-AKT in the hippocampus for each group. The relative protein levels were quantified. *** *p* < 0.001 (vs. control); # *p* < 0.001 (asiatic acid + KA vs. KA); *n* = 5 rats per group.

Calpains are calcium-dependent proteases involved in neuronal death induced by KA [38,39]. Calpain inhibition through calpastatin, the endogenous inhibitor of calpains, can protect against KA-induced hippocampal neuronal damage and cognitive dysfunction [40]. Therefore, we examined the effect of asiatic acid on the expression of calpains and calpastatin in KA-treated rats. As shown in Figure 4A, 72 h after KA administration, the calpain level in the hippocampus was significantly higher in the KA-treated group than in the control group (*p* < 0.001). The expression of calpains in the hippocampus was significantly decreased in the group pretreated with asiatic acid compared with the KA-treated group (calpain 1, *F* (3,16) = 59.1, *p* < 0.001; calpain 2, *F* (3,16) = 21.7, *p* < 0.001). By contrast, the calpastatin level was decreased in the hippocampus of the KA-treated group compared with the control group (*p* < 0.001); however, the decreased levels were reversed by pretreatment with asiatic acid compared with the KA group (*F* (3,16) = 19.6, *p* < 0.001; Figure 4A). In addition, we examined whether asiatic acid treatment contributed to the upregulation of survival signaling through AKT, resulting in neuronal cell survival [41,42]. As shown in Figure 4B, the phosphorylation of AKT (p-AKT) decreased in the hippocampus of the KA-treated group compared with the control group (*p* < 0.001). Asiatic acid pretreatment restored the decreased p-AKT level (*F* (3,16) = 22.4, *p* < 0.001).

### 3.5. Asiatic Acid Preserved the Levels of Synaptic Proteins in the Hippocampus of Rats with KA-Induced Seizures

**Figure 5:**
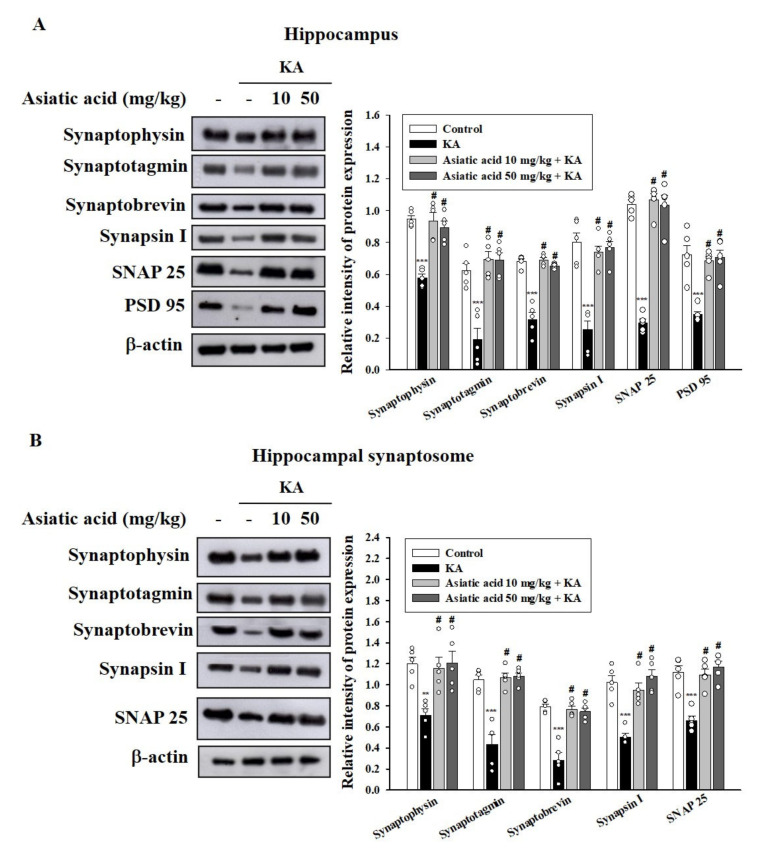
Effects of asiatic acid pretreatment on the levels of synaptic proteins in the hippocampus of rats with KA-induced seizures. Western blot showing the expression levels of synaptophysin, synaptobrevin, synaptotagmin, synapsin-1, and SNAP-25 (synaptosomal-associated protein 25 kDa) in the hippocampal tissues (**A**) and hippocampal synaptosomes (**B**) for each group. The relative protein levels were quantified. ** *p* < 0.01, *** *p* < 0.001 (vs. control); # *p* < 0.001 (asiatic acid + KA vs. KA); *n* = 5 rats per group.

Synaptic proteins, a marker of synaptic activity and plasticity, play a key role in maintaining cognitive function, and their levels have been demonstrated to be decreased in KA-induced excitotoxic injury [23,43]. Therefore, we examined the levels of several synaptic marker proteins in the hippocampus of rats with KA-induced seizures. As shown in Figure 5A, the levels of presynaptic proteins (synaptophysin, synaptotagmin, synaptobrevin, synapsin-1, and SNAP-25) were decreased in the hippocampus of the KA-treated group compared with the control group (*p* < 0.001). Rats pretreated with asiatic acid exhibited significantly higher levels of presynaptic proteins compared with the KA-treated rats (synaptophysin, *F* (3,16) = 23.1, *p* < 0.001; synaptotagmin, *F* (3,16) = 20.1, *p* < 0.001; synaptobrevin, *F* (3,16) = 53.4, *p* < 0.001; synapsin-1, *F* (3,16) = 23.3, *p* < 0.001; SNAP-25, *F* (3,16) = 82.9, *p* < 0.001). The level of the postsynaptic protein PSD-95 in the hippocampus also demonstrated a significant decline in the KA-treated group compared with the control group (*p* < 0.001). These decreases were reversed by asiatic acid pretreatment (*F* (3,16) = 15.1, *p* < 0.001; Figure 5A). In addition, the expression levels of the presynaptic proteins were markedly decreased in the hippocampal synaptosomes of the KA-treated group compared with the control group (*p* < 0.001). Pretreatment with asiatic acid significantly restored the expression of these proteins compared with that in the KA-treated group (synaptophysin, *F* (3,16) = 7.2, *p* < 0.01; synaptotagmin, *F* (3,16) = 28.3, *p* < 0.001; synaptobrevin, *F* (3,16) = 28.8, *p* < 0.001; synapsin-1, *F* (3,16) = 18.8, *p* < 0.001; SNAP-25, *F* (3,16) = 18.4, *p* < 0.001; Figure 5B).

### 3.6. Asiatic Acid Prevented the Decline in Synaptic Vesicles and Mitochondrial Morphology Damage in the Hippocampal Synaptosomes of Rats with KA-Induced Seizures

**Figure 6:**
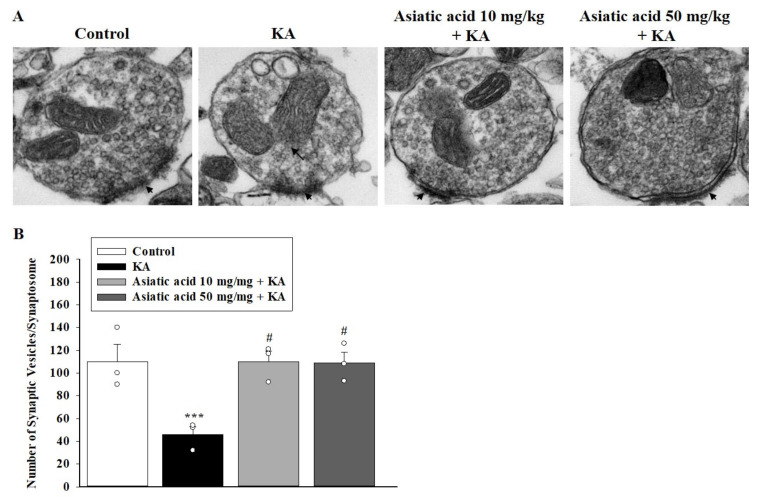
(**A**) Representative transmission electron micrographs of the hippocampal synaptosomal ultrastructure. Each synaptosome contains mitochondria, numerous synaptic vesicles, and a synaptic junction with postsynaptic density (arrowhead). Severe mitochondrial swelling accompanied by a disruption in membrane integrity (arrows). Scale bar, 200 nm. (**B**) The number of synaptic vesicles in the hippocampal synaptosomes was counted. *** *p* < 0.001(vs. control); # *p* < 0.001 (asiatic acid + KA vs. KA); *n* = 3 rats per group.

Because we observed a decline in the levels of presynaptic proteins under seizure and cognitive dysfunction induced by KA, we used transmission electron microscopy to examine the ultrastructure of the hippocampal nerve terminals (synaptosomes). Figure 6A shows that the hippocampal synaptosome of the control group contained mitochondria, numerous synaptic vesicles, and a synaptic junction with postsynaptic density. By contrast, decreased synaptic vesicles and mitochondrial swelling and disruption were observed in the hippocampal synaptosome of KA-treated rats. In rats pretreated with asiatic acid, the KA-caused synaptic vesicle reduction and the mitochondrial structural damage was ameliorated. As shown in Figure 6B, we observed a significant decrease in the number of synaptic vesicles per synaptosome in the KA group compared with the control group (*p* < 0.01); however, the no difference in the number of synaptic vesicles per synaptosome was observed between the asiatic acid groups and the control group (*p* = 1). We noted a significant increase in the number of synaptic vesicles in the groups pretreated with asiatic acid in comparison with the KA-treated group (*F* (3,8) = 8.9, *p* < 0.01).

### 3.7. Proteomic Analysis Identified 12 Proteins Associated with the Effect of Asiatic Acid on the Hippocampal Synaptosomes of Rats with KA-Induced Seizures

**Figure 7:**
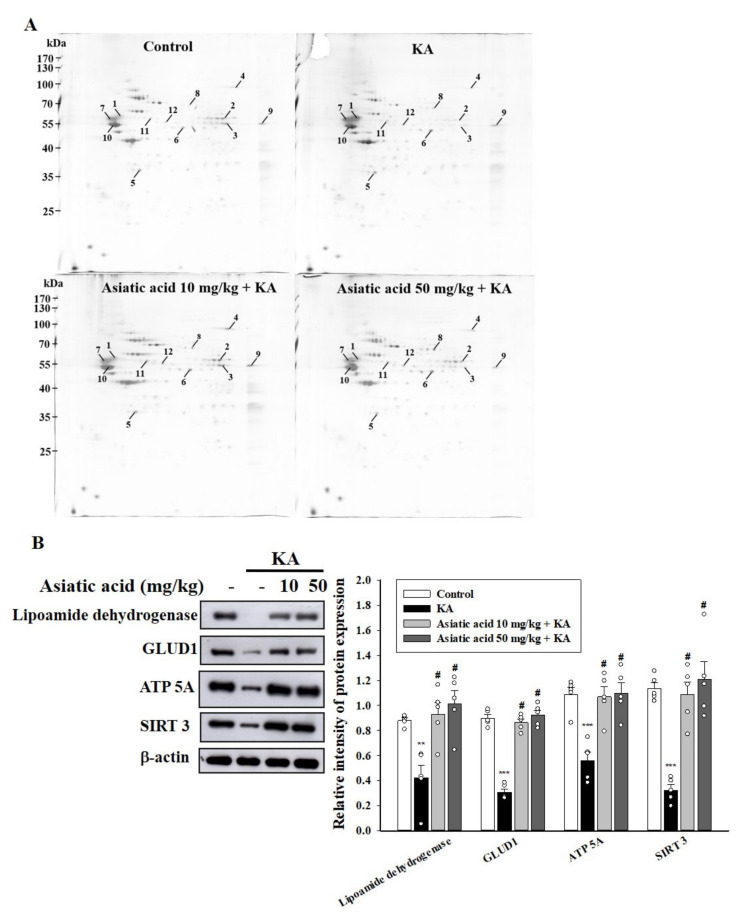
(**A**) Proteomic analysis identified 12 proteins associated with the asiatic acid pretreatment in the hippocampal synaptosomes of rats with KA-induced seizures. Global view of the protein spots on the two-dimensional gels. Protein spots with meaningful changes in intensity are labeled with Arabic numerals. (**B**) Western blot showing the expression levels of lipoamide dehydrogenase, GLUD1 (glutamate dehydrogenase 1), ATP5A (mitochondrial membrane ATP synthase), and SIRT3 (mitochondrial deacetylase sirtuin-3) in the hippocampus for each group. The relative protein levels were quantified. ** *p* < 0.01, *** *p* < 0.001 (vs. control); # *p* < 0.001 (asiatic acid + KA vs. KA); *n* = 5 rats per group.

To explore the specific proteins and potential mechanisms underlying the asiatic acid-mediated cognitive improvement in rats with KA-induced seizures, we performed a proteomic analysis of the hippocampal synaptosomes to identify the proteins responsive to asiatic acid treatment. As shown in Figure 7A, approximately 780 protein spots appeared in each gel, and the MS analysis clearly identified significant changes in the expression levels of 12 proteins (labeled with Arabic numerals in Table 1). Moreover, a group of mitochondrial proteins was identified, including dihydrolipoyl dehydrogenase, glutamate dehydrogenase 1 (GLUD1), aconitatehydratase, and ATP synthase subunits, which are majorly associated with energy generation and various metabolic pathways. In addition, the results of the Western blot analysis revealed that the levels of lipoamide dehydrogenase, GLUD1, ATP5A, and mitochondrial deacetylase SIRT3 decreased in the hippocampal synaptosomes of the KA-treated group compared with the control group (*p* < 0.001). Rats pretreated with asiatic acid exhibited significantly higher levels of lipoamide dehydrogenase, GLUD1, AT5A, and SIRT3 than did rats treated with KA alone (lipoamide dehydrogenase, *F* (3,16) = 9.3.7, *p* < 0.01; GLUD1, *F* (3,16) = 93.1, *p* < 0.001; ATP5A, *F* (3,16) = 12.9, *p* < 0.001; SIRT3, *F* (3,16) = 20.1, *p* < 0.001; Figure 7B)

### 3.8. Functional Network Analysis

**Figure 8:**
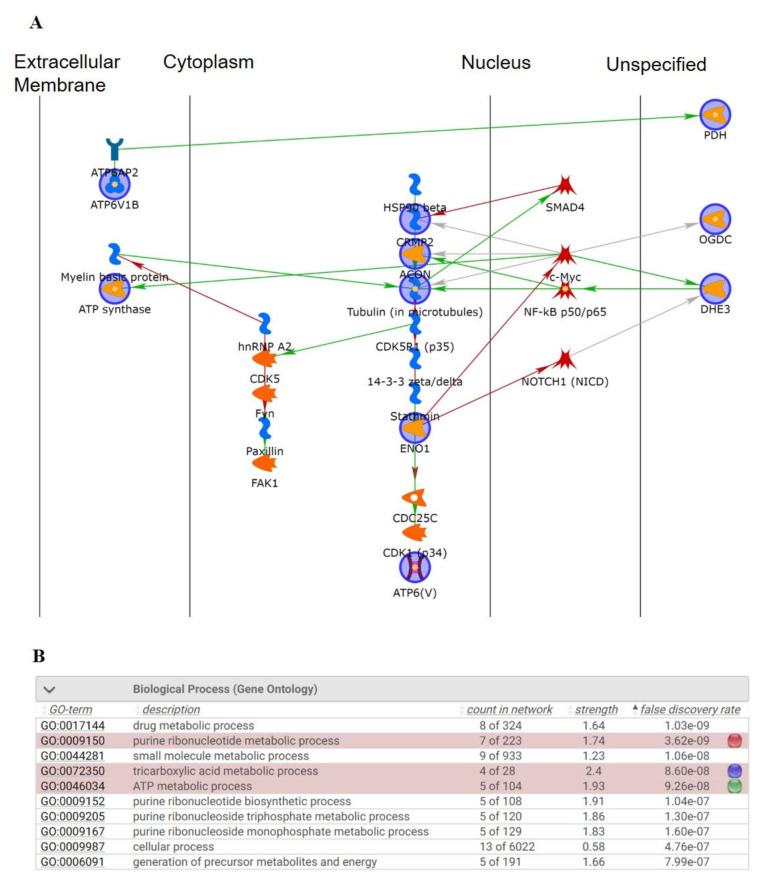
(**A**) Biological network analyses of the differentially expressed proteins using MetaCore™ mapping tools. The nodes represent proteins and the lines between the nodes indicate direct protein–protein interactions. The various proteins on this map are indicated by different symbols representing the functional class of the proteins. (**B**) The top-ranked pathways from the GeneGo MetaCore™ pathway analysis. The pathways were ranked according to the *p* values. Color nodes: query proteins and first shell of interactors.

To delineate the relationships between the differentially expressed proteins elucidated by 2-DE and their significance in the mechanisms associated with the antiepileptic effect of asiatic acid, we used MetaCore™software (Joseph, MI, USA) to predict the interactions among the targeted proteins revealed by the proteomic analysis. The network was generated using the shortest-path algorithm to map the interactions between the proteins. The highlighted lines represent specific, designated pathways, and the background lines represent the secondary, related biological pathways (Figure 8A). On the basis of this network, we found that treatment with asiatic acid affected inflammatory responses, ATP synthesis, and drug metabolism. As shown in Figure 8B, protein–protein interaction networks indicated that the proteins differentially expressed after treatment with asiatic acid were primarily involved in the following processes: drug metabolic process (*p* = 1.03 × 10^−9^), purine ribonucleotide metabolic process (*p* = 3.62 × 10^−9^), tricarboxylic acid metabolic process (*p* = 8.62 × 10^−8^), and ATP metabolic process (*p* = 9.28 × 10^−8^).

## 4. Discussion

Cognitive impairment is frequently observed in patients with epilepsy. Current AEDs only help with seizures and do not improve cognitive impairment [4,6]. Therefore, natural therapeutic agents that can not only treat epilepsy but also improve cognitive impairment must be developed [22,23,44,45]. The findings of the present study indicated that asiatic acid is a novel therapeutic intervention that can ameliorate seizures and associated memory impairment. In the present study, we found that asiatic acid administered before the induction of seizures by KA exhibited anti-seizure activity, improved cognitive function, attenuated neuronal damage, and restored synaptic protein levels and mitochondrial function.

Systemic administration of KA in animals has been widely used as an experimental model for the investigation of epilepsy [26]. Similar to the findings of previous studies [23,46,47], the results of the present study indicated that rats treated with KA developed seizures and memory impairment. Pretreatment with asiatic acid (10 or 50 mg/kg, i.p.) delayed the onset of seizure and reduced the severity of the seizure. This finding is in line with that reported by Wang et al., who revealed that 20–40 mg/kg asiatic acid exhibited anticonvulsant activity in a mouse model of KA-induced seizure [15]. In our study, pretreatment with asiatic acid improved memory impairment in rats with KA-induced seizure, indicating the effectiveness of this natural compound in epilepsy-associated cognitive impairment. Our finding is in agreement with those of previous studies that have reported that asiatic acid improved cognitive function in numerous animal models [10,11,16,48].

Elucidating the possible mechanisms underlying the cognitive improvement by asiatic acid in KA-treated rats is critical. Numerous studies have indicated that neuronal loss in the hippocampus might be a consequence and a cause of cognitive deficits [37,49,50]. In the present study, KA treatment in rats caused significant neuronal death or degeneration in the CA1 and CA3 regions of the hippocampus; this finding is consistent with previous reports [21,22,25]. However, pretreatment with asiatic acid markedly attenuated neuronal loss and degeneration, indicating that asiatic acid protected hippocampal neurons against neurotoxicity in rats with KA-induced seizures. In addition, KA treatment results in neuronal damage or death, with associated increased levels of calpains, which are ubiquitously expressed calcium-activated proteases [39,51]. Calpain inhibition has been demonstrated to improve neuronal function and limit neuronal damage in several excitotoxic brain injuries [38,40,44]. In the current study, we observed that KA treatment increased the level of calpains and reduced the level of calpastatin, a calpain inhibitor, in the hippocampus. These KA-induced alterations were restored by pretreatment with asiatic acid, suggesting that asiatic acid treatment inhibited calpain activation. Moreover, we observed that KA reduced the levels of active AKT, a well-known cell survival pathway, in the hippocampus, which is consistent with the findings of previous studies [41,52]; however, pretreatment with asiatic acid restored the AKT level. Consistent with our findings, previous studies have reported that asiatic acid protects against neuronal damage by increasing AKT activation [14,53]. Thus, we speculate that asiatic acid increased AKT activation and inhibited calpain activation to significantly control neuronal death and protect neuronal cells, thus maintaining cognitive function in rats with KA-induced seizure.

A synaptic decline and plasticity deficiency have been implicated in KA-elicited cognitive impairment [23,43,47]. In the current study, KA treatment in rats reduced the levels of synapse-associated proteins, including presynaptic proteins (synaptobrevin, synapsin-1, and SNAP-25) and the postsynaptic protein PSD-95, and pretreatment with asiatic acid reversed such alterations. These proteins play a crucial regulatory role in the synaptic structure and transmission [54,55]. Particularly, synaptobrevin, synapsin-1, and SNAP-25 are synaptic vesicle-associated proteins that regulate the release of neurotransmitters. Decreases in these proteins and consequent decreases in available synaptic vesicles have been reported to be involved in cognitive dysfunction [56,57]. Therefore, asiatic acid might exert preventive effects against KA-induced cognitive impairment by maintaining the levels of synaptic proteins and vesicles in the hippocampus. This hypothesis is supported by the ultrastructure images of hippocampal synaptosomes, which indicates that pretreatment with asiatic acid restored synaptic vesicles reduced by KA administration. However, how asiatic acid restores the levels of synaptic proteins and vesicles to normal levels in KA-treated rats remains unclear. Calpain activation causes the proteolytic degradation of axonal proteins that play crucial roles in the regulation of neuronal activity [58]. Because asiatic acid inhibited the KA-induced increase in calpain expression in the hippocampus in our study, we speculate that asiatic acid could inhibit the calpain-mediated cleavage of synaptic proteins and consequently preserve synaptic proteins and vesicles in the hippocampus, thus contributing to the improvement of cognitive function of rats with KA-induced seizure.

Adequate energy supply by mitochondria in the brain is essential for neuronal function and survival. Deteriorated mitochondrial function and a consequent decrease in ATP production have been implicated in KA-induced neuronal damage and cognitive deficits [59,60,61]. In the present study, we observed significant mitochondrial ultrastructural damage in the hippocampal synaptosomes of KA-treated rats with seizure; however, pretreatment with asiatic acid prevented such ultrastructural damage. Furthermore, the findings of the proteomic analysis of hippocampal synaptosomes revealed 12 proteins associated with the effect of asiatic acid, particularly its effects on the mitochondrial proteins related to energy generation. Consistent with the results of the proteomics analysis, the levels of presynaptic mitochondrial proteins, namely lipoamide dehydrogenase, GLUD1, ATP synthase, and SIRT3, were significantly upregulated by pretreatment with asiatic acid. Moreover, our functional network analysis results revealed a relationship between asiatic acid and metabolic processes, especially the ATP metabolic process. In fact, energy is crucial for the brain to signal normally, while loss of energy can contribute to seizure generation by destabilizing membrane potentials and signaling in the chronic epileptic brain [62,63]. Furthermore, it has been shown that presynaptic mitochondria affect the magnitude of synaptic vesicle exocytosis, primarily through ATP synthesis [64]. Thus, we speculated that asiatic acid might increase synaptic vesicle exocytosis by alleviating KA-induced presynaptic mitochondrial damage and increasing ATP production in hippocampal neurons, which may be crucial to maintain synaptic function and prevent cognitive deficits. However, the details regarding the mechanism of asiatic acid’s modulation of presynaptic mitochondrial bioenergetics were not elucidated in this study, and that should be investigated further.

Numerous studies have highlighted the cognitive improvement of asiatic acid [10,11,16,48]. Although the mechanism by which asiatic acid exhibits this beneficial effect is still under investigation, maintained anti-oxidative activity, suppressed neuroinflammation, increased acetylcholine synthesis, and promoted neurogenesis have been proposed [65]. In the present study, we did not examine the anti-inflammatory effect of asiatic acid in KA-injected rats. However, inflammatory responses, such as glia activation and inflammatory cytokine production, has been described in human epilepsy and in experimental models of epilepsy [66,67]. Thus, the possible involvement of suppressing inflammatory processes in the neuroprotective effect of asiatic acid observed in the present study should be considered.

**Figure 9:**
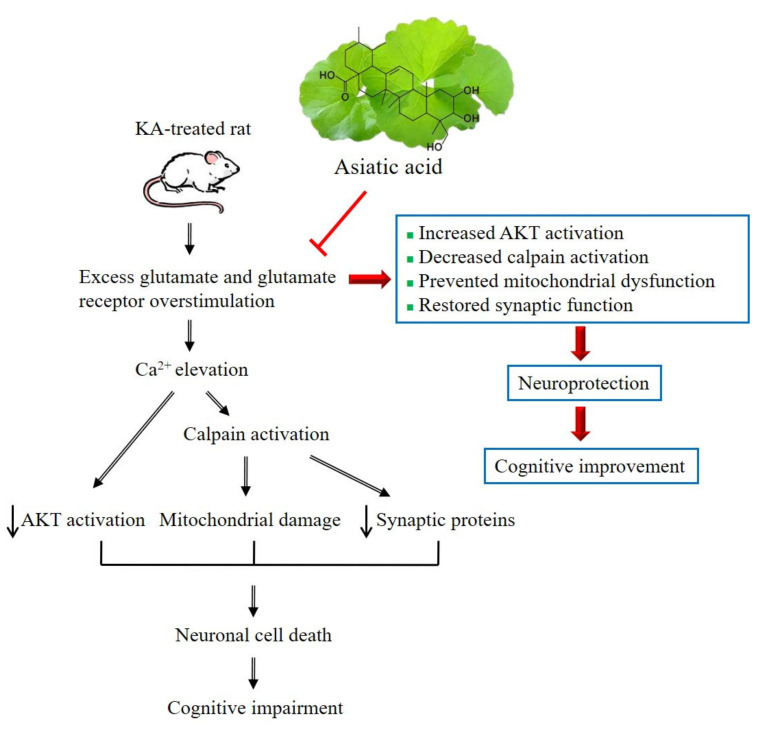
The proposed possible mechanisms underlying the cognitive improvement by asiatic acid in rats with KA-induced seizures. KA causes excessive glutamate release and consequent glutamate receptor overstimulation, resulting in Ca^2+^ elevation, decreased AKT activation, increased calpain activation, decreased synaptic proteins, mitochondrial damage, and eventually neuronal damage and death, which may contribute to cognitive deficits. Asiatic acid, through inhibiting glutamate release, can effectively inhibit calpain activation, increase AKT activation, and preserve synaptic and mitochondrial function, thus contributing to the improvement of the cognitive dysfunction of rats with KA-induced seizures.

KA causes excessive glutamate release and consequent glutamate receptor overstimulation, resulting in Ca^2+^ elevation, decreased AKT activation, and increased calpain activation. Decreased AKT or increased calpain activity results in the proteolytic degradation of synaptic proteins and mitochondrial damage and eventually neuronal damage and death, which may contribute to cognitive deficits [40,47,52,58,61]. Our results, together with the inhibition of glutamate release by asiatic acid [36], suggest asiatic acid may exert preventive effects against cognitive deficits in rats with KA-induced seizures through inhibiting glutamate release to increase AKT activation, inhibit calpain activation, and preserve synaptic and mitochondrial function (Figure 9).

The limitation of this study is that we do not know how much asiatic acid actually enters the brain. It has been reported that the therapeutic efficacy of asiatic acid is restricted due to its poor solubility and low penetrability into the brain [68]. Since brain permeability is a crucial factor for a drug to exhibit therapeutic effects at a target site, further study is warranted to elucidate the penetrability of asiatic acid in an in vivo, KA-induced cognitive-deficit model. However, it is known that in vivo animal models, while extremely useful in unravelling the mechanisms of human physiology, have limited capacity to mimic the complex and dynamic microenvironment of the human organs. To overcome these limitations, several 3-dimensional (3D) microfluidic and BBB on-a-chip and brain microvessel models have been developed. These models, which are capable of self-organization and functionality similar to the tissue of origin, can provide an alternative for drug analysis and development [69]. Although a successful result was achieved in this study, more studies are needed to assess whether asiatic acid treatment in humans is feasible.

## 5. Conclusions

Pretreatment with asiatic acid abrogates KA-induced pathophysiological events, such as neuronal cell death, decreased synaptic protein levels, and mitochondrial dysfunction in the hippocampus; these effects may at least partly contribute to the improvement of cognitive dysfunction. Therefore, asiatic acid may be a beneficial natural product for the treatment of epilepsy and associated cognitive deficits.

## Figures and Tables

**Table 1:** List of identified protein spots.

Spot No.	Protein Name	Accession Number	Mw/pI	Score (Coverage)	Match Fragment	Subcellular Location	Function
1	Tubulin alpha-1A chain	P68370	50.788/4.94	69 (35%)	9	Cytoplasm, cytoskeleton.	Tubulin is the major constituent of microtubules. It binds two moles of GTP, one at an exchangeable site on the beta chain and one at a non-exchangeable site on the alpha chain.
2	Dihydrolipoyl dehydrogenase	Q6P6R2	54.574/7.96	113 (27%)	12	Mitochondrion matrix	Lipoamide dehydrogenase is a component of the glycine cleavage system as well as an E3 component of three alpha-ketoacid dehydrogenase complexes (pyruvate-, alpha-ketoglutarate-, and branched- chain amino acid-dehydrogenase complex).
3	Glutamate dehydrogenase 1	P10860	61.719/8.05	89 (28%)	12	Mitochondrion Endoplasmic reticulum	Mitochondrial glutamate dehydrogenase that converts L- glutamate into alpha-ketoglutarate. Plays a key role in glutamine anaplerosis by producing alpha-ketoglutarate, an important intermediate in the tricarboxylic acid cycle (By similarity). May be involved in learning and memory reactions by increasing the turnover of the excitatory neurotransmitter glutamate (PubMed:9275181).
4	Aconitate hydratase	Q9ER34	86.121/7.87	159 (29%)	16	Mitochondrion	Catalyzes the isomerization of citrate to isocitrate via cis-aconitate.
5	Pyruvate dehydrogenase E1 component subunit beta	P49432	39.299/6.20	130 (46%)	12	Mitochondrion matrix	The pyruvate dehydrogenase complex catalyzes the overall conversion of pyruvate to acetyl-CoA and CO_2_, and thereby links the glycolytic pathway to the tricarboxylic cycle.
6	Alpha-enolase	P04764	47.44/6.16	223 (56%)	22	Cytoplasm. Cell membrane.	Glycolytic enzyme the catalyzes the conversion of 2- phosphoglycerate to phosphoenolpyruvate. In addition to glycolysis, involved in various processes such as growth control, hypoxia tolerance and allergic responses. May also function in the intravascular and pericellular fibrinolytic system due to its ability to serve as a receptor and activator of plasminogen on the cell surface of several cell-types such as leukocytes and neurons.
7	Tubulin beta-2A chain	P85108	50.274/4.78	290 (59%)	29	Cytoplasm, cytoskeleton	Tubulin is the major constituent of microtubules. It binds two moles of GTP, one at an exchangeable site on the beta chain and one at a non-exchangeable site on the alpha chain (By similarity).
8	Dihydropyrimidinase-related protein 2	P47942	62.638/5.95	217 (45%)	20	Cytoplasm	Plays a role in neuronal development and polarity, as well as in axon growth and guidance, neuronal growth cone collapse and cell migration. Necessary for signaling by class 3 semaphorins and subsequent remodeling of the cytoskeleton.
9	ATP synthase subunit alpha	P15999	59.831/9.22	205 (45%)	22	Mitochondrion	Mitochondrial membrane ATP synthase (F_1_F_0_ ATP synthase or Complex V) produces ATP from ADP in the presence of a proton gradient across the membrane which is generated by electron transport complexes of the respiratory chain.
10	ATP synthase subunit beta	P10719	56.318/5.19	266 (64%)	33	Mitochondrion inner membrane	During catalysis, ATP synthesis in the catalytic domain of F_1_ is coupled via a rotary mechanism of the central stalk subunits to proton translocation. Subunits alpha and beta form the catalytic core in F_1_.
11	V-type proton ATPase subunit B	P62815	56.857/5.57	276 (61%)	26	Membrane	Non-catalytic subunit of the peripheral V1 complex of vacuolar ATPase. V-ATPase is responsible for acidifying a variety of intracellular compartments in eukaryotic cells.
12	Dihydrolipoyllysine-residue succinyltransferase component of 2-oxoglutarate dehydrogenase complex	Q01205	49.236/8.89	75 (20%)	8	Mitochondrion matrix	The 2-oxoglutarate dehydrogenase complex catalyzes the overall conversion of 2-oxoglutarate to succinyl-CoA and CO_2_ (By similarity). The 2-oxoglutarate dehydrogenase complex is mainly active in the mitochondrion.

## Data Availability

Not applicable.
